# High resolution crystal structure of the catalytic domain of MCR-1

**DOI:** 10.1038/srep39540

**Published:** 2016-12-21

**Authors:** Guixing Ma, Yifan Zhu, Zhicheng Yu, Ashfaq Ahmad, Hongmin Zhang

**Affiliations:** 1 Department of Biology and Shenzhen Key Laboratory of Cell Microenvironment, Southern University of Science and Technology, Shenzhen 518055, China

## Abstract

The newly identified mobile colistin resistant gene (*mcr-1*) rapidly spread among different bacterial strains and confers colistin resistance to its host, which has become a global concern. Based on sequence alignment, MCR-1 should be a phosphoethanolamine transferase, members of the YhjW/YjdB/YijP superfamily and catalyze the addition of phosphoethanolamine to lipid A, which needs to be validated experimentally. Here we report the first high-resolution crystal structure of the C-terminal catalytic domain of MCR-1 (MCR-1C) in its native state. The active pocket of native MCR-1C depicts unphosphorylated nucleophilic residue Thr285 in coordination with two Zinc ions and water molecules. A flexible adjacent active site loop (aa: Lys348-365) pose an open conformation compared to its structural homologues, suggesting of an open substrate entry channel. Taken together, this structure sets ground for further study of substrate binding and MCR-1 catalytic mechanism in development of potential therapeutic agents.

Since the clinical introduction of penicillin in the 1940s, antibiotics have remained the first choice for treatment against bacterial infections. Emergence of bacterial pathogens with acquired resistance to most of the available antimicrobial agents, namely ‘superbugs’, has greatly restricted the therapeutic choices in last decade^1^. It has become a challenge to treat infections caused by Gram-negative bacteria, such as *Pseudomonas aeruginosa* and *Acinetobacter baumannii*^2^,^3^. Nowadays, many of the pathogenic and common non-pathogenic bacteria harbor antibiotic resistance genes^4^, that is spreading and has been a global nuisance.

Infections caused by Gram negative bacteria are primarily treated by β-lactam antibiotics, such as penicillins, cephalosporins and carbapenems. However, the acquired β-lactam hydrolysing genes like metallo-β-lactamase (*bla*NDM-1)^5^ and *Klebsiella pneumoniae* carbapenemase (*bla*KPC)^6^ have limited the efficacy of β-lactam antibiotics^5^,^7^,^8^,^9^,^10^,^11^. Cationic polypeptides like polymyxin B and colistin (polymyxin E) are regarded as the most effective weapons to combat β-lactam resistant bacteria infections^12^. Structurally and pharmacologically similar polymyxin B and colistin have shown broad-spectrum activities against Gram negative bacteria. However, the rapid spread of resistant bacteria accelerate the global use of colistin in both clinical settings and animal production^13^, which will inevitably give rise to colistin resistant bacteria eventually^12^.

Recently, Liu *et al*. reported a plasmid-borne colistin resistant gene (*mcr-1*) from China^14^, which was quickly reaffirmed in strains isolated from Denmark^15^, Germany^16^, France^17^, Netherland^18^, Switzerland^19^, United States^20^, Belgium^21^, Italy^22^ and Vietnam^23^. This indicates global spreading of bacteria harboring this plasmid-borne colistin resistance. Further, in contrast to early reported chromosome-borne polymyxin resistant genes^24^,^25^,^26^, *mcr-1* can be quickly transmitted among bacterial community through horizontal transfer. Therefore, transfer of colistin resistance to multidrug resistant *Enterobacteriaceae* that already harbor *bla*NDM-1 or *bla*KPC-2 is of a particular concern. Surprisingly, strains holding both resistant genes (*mcr-1* and *bla*NDM-9, *mcr-1.2* and *bla*KPC-3) have been rapidly identified in many recent studies^16^,^19^,^21^,^27^,^28^,^29^,^30^,^31^.

Based on sequence alignment, MCR-1 should be a phosphoethanolamine transferase, a member of YhjW/YjdB/YijP superfamily^32^ which catalyzes the addition of phosphoethanolamine to lipid A moiety of lipopolysaccharides (LPS) and therefore confers colistin resitance to its host^33^,^34^. MCR-1 homologues LptA and EptC can modify the 1′ and 4′ phosphoryl groups of lipid A using phosphoethanolamine (PEA) as a substrate. LptA can specifically transfer PEA to only lipid A phosphoryl groups^32^ while EptC shows a much broader substrate tolerance, such as 1′ and 4′ phosphoryl groups of lipid A, the first heptose residue of the inner-core oligosaccharide of lipooligosaccharide (LOS), residue Thr75 of the flagellar rod protein FlgG and the N-linked glycans of numerous glycoproteins^35^,^36^,^37^,^38^. However, the substrate preference of MCR-1 remains un-characterized to date. To understand the molecular mechanism of colistin resistance and structural features, here we report the X-ray structure of the C-terminal catalytical domain of MCR-1 (aa: 219–541, MCR-1C for short) determined at 1.45 Å resolution. Our results illustrated the detailed structure of MCR-1C which will help in the understanding of the catalysis mechanism of MCR-1 and potential drug design against colistin resistance.

## Results

### Overall structure of MCR-1

MCR-1 contains two domains, one N-terminal transmembrane domain and one periplasmic C-terminal catalytic domain^33^. Several attempts were made to express full-length MCR-1 that resulted in insoluble protein whereas two truncations (aa: S181-R541 and T219-R541) were successfully expressed and crystallized. Crystals of the longer and shorter truncations diffracted to 2.2 Å and 1.45 Å, respectively. Thus, the shorter truncation was used for structure determination whereas the longer one was considered for ICP-MS analysis. The protein was crystallized in space group P2_1_2_1_2_1_ having one MCR-1C molecule in the asymmetric unit with a solvent content of 42.5%. The structure was solved by single-wavelength anomalous scattering (SAD) method using the anomalous signal of zinc ions with well-defined electron density map for all the residues in the construct.

Several structural homologues from hydrolase superfamily are identified via online DALI server^39^ including lipooligosaccharide phosphoethanolamine transferase A from *Neisseria gonorrhoeae* (LptA, PDB: 4KAV and 4KAY)^33^, phosphoethanolamine transferase C from *Campylobacter jejuni* (EptC, PDB: 4TN0)^34^, choline sulfatase (betC, PDB: 4UG4)^40^ and phosphonate monoester hydrolase (PMH, PDB: 2W8S)^41^. The nearest structural homologues are LptA and EptC with sequence identities of 40–41% whereas betC and PMH are distant homologues having sequence identities of 16% and 13%, respectively.

**Figure 1:**
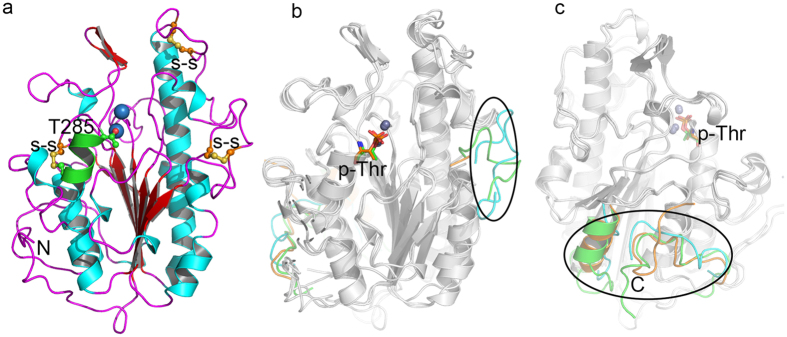
Overall structure of MCR-1C and comparison with LptA and EptC. MCR-1C, LptA (PDB: 4KAY), EptC (PDB: 4TN0) are shown in cartoon representation. The labeled key residues and active site metal ions are represented in ball-and-stick and sphere, respectively. (**a**) Overall structure of MCR-1C. The nucleophilic residue T285 and three disulfide bridges are shown as ball-and-stick. The helix locked by a disulfide bond to the central β sheet is shown in green. (**b,c**) Structural superimpositions (front and back view) of MCR-1C, LptA and EptC are shown in grey. The significant variable loops are highlighted in cyan (MCR-1C), green (LptA) and orange (EptC).

The MCR-1C adopted an α/β/α topology with seven β-strands, sandwiched between two layers of α-helices (Fig. 1a). Based on structural superimposition, MCR-1C is similar to its homologues LptA and EptC (Fig. 1b,c) with a r.m.s.d of 1.58 Å and 1.56 Å (all C-alpha), respectively. We observed that the central β sheets topology is well-superimposed to LptA and EptC while loops adjacent to the active site and the C-terminal fragment are variable (Fig. 1b,c). The conformational variations of the loops adjacent to the active site may infer to the substrate specificity of these enzymes. During review of this manuscript, the other crystal structure of MCR-1 (the C-terminal domain of MCR-1 from residue D218 to residue R541, cMCR-1) was released with PDB code 5K4P^42^. Superposition of current structure to cMCR-1 (5K4P) gives an r.m.s.d value of only 0.49 Å for 323 C-alpha atoms, indicating that these two structures are almost identical. The most different region between these two structures is a loop located at the tip of the structure (aa: 416–422).

MCR-1C consists of three disulfide bridges as revealed in the crystal structure (Fig. 1a, shown as ball-and-stick models). In contrast, LptA and EptC retain five and three, respectively. Moreover, only two disulfide bridges, Cys281-Cys291 and Cys414-Cys422 retain their sequence and structural conservation among all the three structures. The Cys414-Cys422 bridge stabilized a loop (aa: Lys409-Glu423) located at the top of the protein whereas Cys281-Cys291 completes the catalytic site by locking a smaller α-helix (aa: Thr285-Met292) to the central β sheet. It is worth mentioning that residue Thr285 is a catalytic residue for all phosphoenthanolamine transferases^33^,^34^,^42^. The formation of disulfide bond Cys281-Cys291 might restrain helical flexibility to accelerate the catalysis reaction. The third disulfide bridge Cys356-Cys364 is only shared between MCR-1C and LptA and it seems to arrest the conformational freedom of the loop (aa: Lys348–365) (Fig. 1b) and facilitate substrate entry. In contrast, there is no such a loop in this region of EptC (Fig. 1b), which may allow the entry of many different substrates. [Table t1]This is consistent with the multiple reactions catalyzed by EptC^34^.

### Putative active site of MCR-1C

To study the putative active site, we over-expressed MCR-1C in *E. coli* BL21(DE3) plysS and the metal ions bound to the protein were characterized by ICP-MS method. In regular LB medium, the protein can bind Fe, Zn, Mg and Mn ions but prefers Zn as compared to other metal ions (Table 2). In a 50 μM ZnCl_2_ supplemented LB medium, MCR-1C can bind up to four zinc ions (Table 2) where three of them can be located in the structure as determined by SHELXD^43^ based on the anomalous signal. The fourth zinc ion might bind the protein non-specifically due to which we cannot identify it in the structure. This is also revealed in the newly published cMCR-1 (5K4P)^42^ where 10 zinc ions were identified in the structure with most of them locate on the surface coordinating to waters with low occupancy.

**Figure 2:**
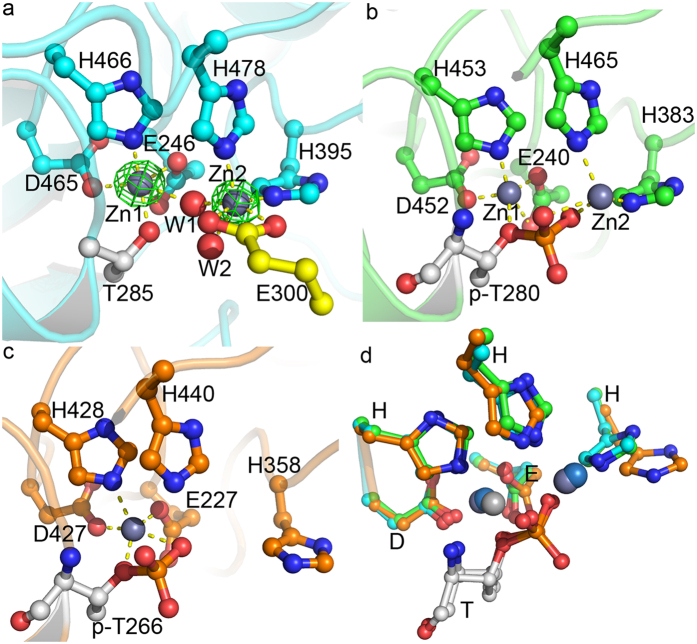
Active site conformations of MCR-1C, LptA and EptC. Active site residues coordinated to metal ions were labeled and shown as ball-and-stick models. Metal ions were shown as balls and coordination bonds were shown as yellow dash lines. (**a**) Active site of MCR-1. Anomalous electron density map was shown for Zinc ions in green mesh at 20σ. Two water molecules involved in Zinc coordination were shown as red balls (W1 and W2). Residue E300 from a neighboring molecule also involved in coordination to Zn2 was shown with yellow carbons. Both Zinc ions were hexa-coordinated to protein residues and water molecules. (**b**) Active site of LptA (PDB code 4KAY). The coordination numbers for Zn1 and Zn2 were five and four, respectively. The phosphate group on phospho-Thr participated in Zinc coordination. (**c**) Active site of EptC. Only one Zinc ion was identified in EptC. The coordination number is five and phosphate group on phospho-Thr also participated in coordination. (**d**) Superposition of the active sites of MCR-1, LptA and EptC. All residues were well superimposed except one histidine residue (His358 in EptC) that involved in the coordination to Zn2 in MCR-1 and LptA.

In our study among the three identified zinc ions in MCR-1C, two of them were located at the active site (Fig. 1a) and the third one located near residue Cys291. Similar to LptA and EptC, the first zinc ion (Zn1) is highly conserved in active site contacts and is coordinated to residues Glu246, Thr285, Asp465 and His466^33^,^34^,^44^ (Fig. 2). Beside the conserved orientations of these coordination residues among MCR-1C, LptA and EptC, we found that unlike LptA, EptC and even cMCR-1^42^, Thr285 (a putative nucleophilic attacking residue in catalysis) is un-phosphorylated. The phosphorylated Thr285 is considered a catalysis intermediate during phosphate transfer^33^,^34^,^42^,^45^ thus stating MCR-1C the first native structure without any bound substrate or reaction intermediate. In contrast, Thr285 in cMCR-1 is phosphorylated and the coordination of Zn1 is very similar to that of LptA.

Like the Zinc-soaked LptA (PDB: 4KAY), the second Zinc ion (Zn2) coordinates to two conserved histidine residues (His395 and His478 in MCR-1C) among some phospho-form transferases. However, the MCR-1C crystals uptake Zn ions from the medium for cell culture rather than soaking, suggesting a natural co-factor which is strong enough not to be washed off during protein purification. Due to un-phosphorylated nature, except the two histidine residues, Zn2 ion coordinates to water molecules and carboxylate moiety of Glu300 from a neighboring MCR-1C (Fig. 2a). The Glu300 interaction might not be observed in solution since MCR-1C is a monomer as determined by multi-angle light scattering method (Fig. S1). Moreover, this coordination could be fulfilled by the incoming substrate and possibly be a part of the catalytic mechanism. Crystals of the newly released cMCR-1 were obtained in a solution containing 0.2 M zinc acetate and Zn2 in this structure coordinates to the phosphate group and only one histidine residue His395, but not His478^33^,^42^, which is quite different from both LptA and our MCR-1C structure and needs further validation.

## Discussion

The newly identified *mcr-1* is a mobile colistin resistant gene and has been shown to rapidly spread among various bacterial strains through horizontal transfer and assisted its host in colistin resistance^14^. Since the broad-spectrum β-lactam antibiotics resistant gene, such as *bla*NDM-1, has spread all over the world, colistin is regarded as an important resort antibiotic against these superbugs^12^. Several studies have reported the presence of *bla*NDM-9 and *mcr-1* in chicken meat samples^27^ including carbapenem and colistin double resistant *E. coli* strains^19^,^29^,^30^. Furthermore, *mcr-1*-harbouring plasmids have been reported in multi-drug resistant *E. coli*^46^,^47^, arising more threat to our healthcare system^1^.

Based on the published reports, it is imperative to study the molecular features and catalysis mechanism of MCR-1. This is the first study that reports the high-resolution crystal structure of the C-terminal catalytical domain of MCR-1 in native state. The overall structure of MCR-1C closely resembles the other two phosphoethanolamine transferases LptA and EptC. The intriguing difference among them is found to be at the loop (aa: Lys348–365) adjacent to the active site and might regulate substrate entry and specificity (Fig. 1b). Interestingly, the absence of this loop in EptC is anticipated to allow entry of different substrates (Fig. 1b). Our notion is supported by the fact that EptC retains the ability of catalyzing multiple reactions^34^.

At the putative active site, the presence of several conserved residues in phosphorylated transferases indicates that MCR-1C might also be a phosphoethanolamine transferase which needs to be confirmed by further biochemical and functional studies. The phosphorylated nature of active site residue Thr285 is considered an intermediate form in phospho-transfer reaction^33^,^34^,^42^,^45^, therefore we believe the unphosphorylated active site of MCR-1C to be in a native state before substrate binding.

The high resolution structure of MCR-1C sets up the primary basis for further study of substrate binding and catalysis mechanism. Future studies are required to search and validate MCR-1 inhibitors and block its enzymatic activities. The crystallographic and biophysical studies reported here lay the groundwork for developing new therapeutic strategies and chemicals against colistin resistant superbugs.

## Materials and Methods

### Protein expression and purification

Gene fragments of *mcr-1* (corresponding to amino acid residue range of 219–541 and 181–541) were synthesized and inserted into plasmid pRHSUL2 (modified in our lab by insetting a His-SUMO tag at the 5′ open reading frame to pRSET A^48^). The plasmid was transferred into *E. coli* BL21(DE3) plysS cells and cultured at 37 °C. Protein expression was induced at cell density of OD_600_ = 0.6~0.8 with 0.5 mM IPTG and 50 μM ZnCl_2_ at 16 °C for 16~18 hrs. The cell pellets were harvested by centrifugation and lysed by sonication in lysis solution (20 mM Tris-HCl pH 8.0, 500 mM NaCl, 0.5% (v/v) TritonX-100). The cell debris was removed by centrifugation and the cleared supernatant was loaded onto a HisTrap HP column (GE Healthcare) mounted on an AKTA pure (GE Healthcare). The column was washed with lysis buffer supplemented with 20 mM and 40 mM imidazole, and the protein was eluted with lysis buffer supplemented with 200 mM imidazole. The His-SUMO tag was cleaved by adding SUMO protease to the eluted protein fraction during dialysis to lower the concentration of imidazole. And then the protein was re-loaded to HisTrap column and the flow-through fraction was collected. The protein was further purified by anion exchange column HiTrap Q HP (GE Healthcare) at pH 8 with a sodium chloride gradient from 0 to 0.5 M. The purified protein was concentrated to 150 mg/ml (measured at OD_280_ with an extinction coefficient of 37735) in 20 mM Tris-HCl pH 8.0 for crystallization trials.

### Crystallization, data collection and structure determination

Crystals of MCR-1 were obtained by the hanging drop vapor diffusion method at 20 °C by mixing 1 μl of purified protein and 1 μl of reservoir solution of 100 mM Tris-HCl pH 8.0 and 20% (v/v) PEG 4000. Columnar crystals appeared in a week. Fresh, single and large crystals of MCR-1 were soaked in 100 mM Tris-HCl pH 8.0, 20% (v/v) PEG 4000 and 16% (v/v) glycerol. The crystals were harvested with a nylon loop and immediately cooled in liquid nitrogen. Excitation scan was performed at beamline BL17U1 at Shanghai Synchrotron Radiation Facility (SSRF) at the Zinc absorption edge to confirm the identity of bound metal ions. The diffraction data were collected and processed using HKL2000^49^. Solvent content and molecule number of MCR-1 per asymmetric unit were calculated using Matthews^50^.

The structure was solved using the SAS protocol of Auto-Rickshaw: the EMBL-Hamburg automated crystal structure determination platform^51^. The input diffraction data were prepared and converted for use in Auto-Rickshaw using programs of the CCP4 suite^52^. FA values were calculated using the program SHELXC^53^. Based on an initial analysis of the data, the maximum resolution for substructure determination and initial phase calculation was set to 1.7 Å. All of the 3 heavy atoms requested were found using the program SHELXD^43^. The correct hand for the substructure was determined using the programs ABS^54^ and SHELXE^55^. Initial phases were calculated after density modification using the program SHELXE. 95.36% of the model was built using the program ARP/wARP^56^,^57^. The model was refined with PHENIX^58^ and Refmac^59^ in CCP4 suite and cycled with rebuilding in COOT^60^. Solvents were added automatically in Coot and then manually inspected and modified. The final model was analyzed with MolProbity^61^, showing that 323 over 331 residues were in the favored region of Ramachandran plot and only one residue in outlier region (Ser330). The electron density map at residue S330 was very clear and there should be no doubt about its conformation. Data collection and model refinement statistics were summarized in Table 1. The coordinates and structure factors were deposited in PDB with entry code 5GRR. Structural figures were drawn with PyMOL^62^.

### Metal ion identification

Metal ions bound by MCR-1 were identified through ICP-MS assays using Orbitrap Fusion Tribrid mass spectrometer (MS, Thermo Scientific) with the LCQ Fleet MS detector (Thermo Scientific) in the core facility of our university. Two different samples of MCR-1 were assayed and the contents of bound metal ions were compared, i.e. MCR-1 expressed in regular LB, MCR-1 expressed in LB supplemented with 50 μM ZnCl_2._ Three independent assays were performed for each sample and Table 2 listed the ion contents for each sample.

## Additional Information

**How to cite this article**: Ma, G. *et al*. High resolution crystal structure of the catalytic domain of MCR-1. *Sci. Rep.* **6**, 39540; doi: 10.1038/srep39540 (2016).

**Publisher's note:** Springer Nature remains neutral with regard to jurisdictional claims in published maps and institutional affiliations.

## Supplementary Material

Supplementary Information

## Figures and Tables

**Table 1 t1:** X-ray data collection and refinement statistics.

Parameters	Data 1	Data 2
**Data collection**		
Space group	*P2*_*1*_*2*_*1*_*2*_*1*_	*P2*_*1*_*2*_*1*_*2*_*1*_
Cell dimensions		
a, b, c (Å)	a = 47.29, b = 62.70, c = 104.82	a = 47.31, b = 62.70, c = 104.83
α, β, γ (°)	α = β = γ = 90°	α = β = γ = 90°
Wavelength (Å)	0.91	0.91
Resolution range (Å)	50-1.55 (1.61–1.55)*	50-1.45 (1.50–1.45)*
No. of all observed reflections	46229	55886
Unique reflections	46163 (4484)	54741 (4348)
Completeness (%)	100.0 (100.0)	100.0 (100.0)
R_merge_ (%)	0.085 (0.733)	0.078 (0.809)
I/σ(I)	34.7 (4.8)	23.6 (2.6)
Redundancy	14.0 (14.1)	7.8 (7.9)
Wilson B factor	12.69	13.45
**Refinement**		
Resolution range (Å)		50–1.45
No. of reflections used		54740 (4348)
Reflections used in R_free_		2715 (228)
R_work_		0.1527 (0.223)
R_free_		0.1767 (0.240)
No. of nonhydrogen atoms		3080
Macromolecules		2573
Zn/Glycerol		3/2
Water		492
Protein residues		323
RMSD (bonds)		0.009
RMSD (angles)		1.44
Ramachandran favored (%)		97.6
Ramachandran allowed (%)		2.1
Ramachandran outliers (%)		0.3
Average B-factor (Å^2^)		23.70
Protein		21.28
Zn ions		15.25
Water		36.27

^*^Values in parentheses are for highest resolution shell.

**Table 2:** ICP-MS assay of the ions contents in MCR-1.

Ions type	Zn	Fe	Mg	Mn	Cr	Co	Cu	Se	Mo	Cd	Pb
Control	0.06 ± 0.07	0.05 ± 0.06	0.12 ± 0.15	<0.01	<0.01	<0.01	<0.01	<0.01	0.18 ± 0.31	<0.01	<0.01
MCR-1C (regular)	1.06 ± 0.61	0.36 ± 0.57	0.14 ± 0.21	0.06 ± 0.11	<0.01	<0.01	<0.01	<0.01	0.01 ± 0.01	<0.01	0.01 ± 0.02
MCR-1C (Zn)	4.02 ± 0.96	0.01 ± 0.01	0.02 ± 0.03	<0.01	<0.01	<0.01	<0.01	<0.01	0.13 ± 0.22	<0.01	<0.01

Metal ion quantities are calculated for per molecule of MCR-1C, and using 50 mM Tris (pH 8.0) buffer as control. MCR-1C (regular) was expression in regular LB medium and MCR-1C (Zn) was expression in LB medium containing 50 μM ZnCl_2_.
