# Supplementary material for: High resolution crystal structure of the catalytic domain of MCR-1
Source: Sci Rep. 2016 Dec 21;6:39540. doi: 10.1038/srep39540 (PMC5175174; doi:10.1038/srep39540)
Supplement: Supplementary Information [file srep39540-s1.pdf]

High resolution crystal structure of the catalytic domain of MCR-1

Guixing Ma[1], Yifan Zhu[1], Zhicheng Yu[1], Ashfaq Ahmad[1], Hongmin Zhang[1*]

[1]Department of Biology and Shenzhen Key Laboratory of Cell Microenvironment, Southern University of Science and Technology, Shenzhen 518055, China.

[*]Correspondence should be addressed to Hongmin Zhang

(hongmin_zhang@foxmail.com)

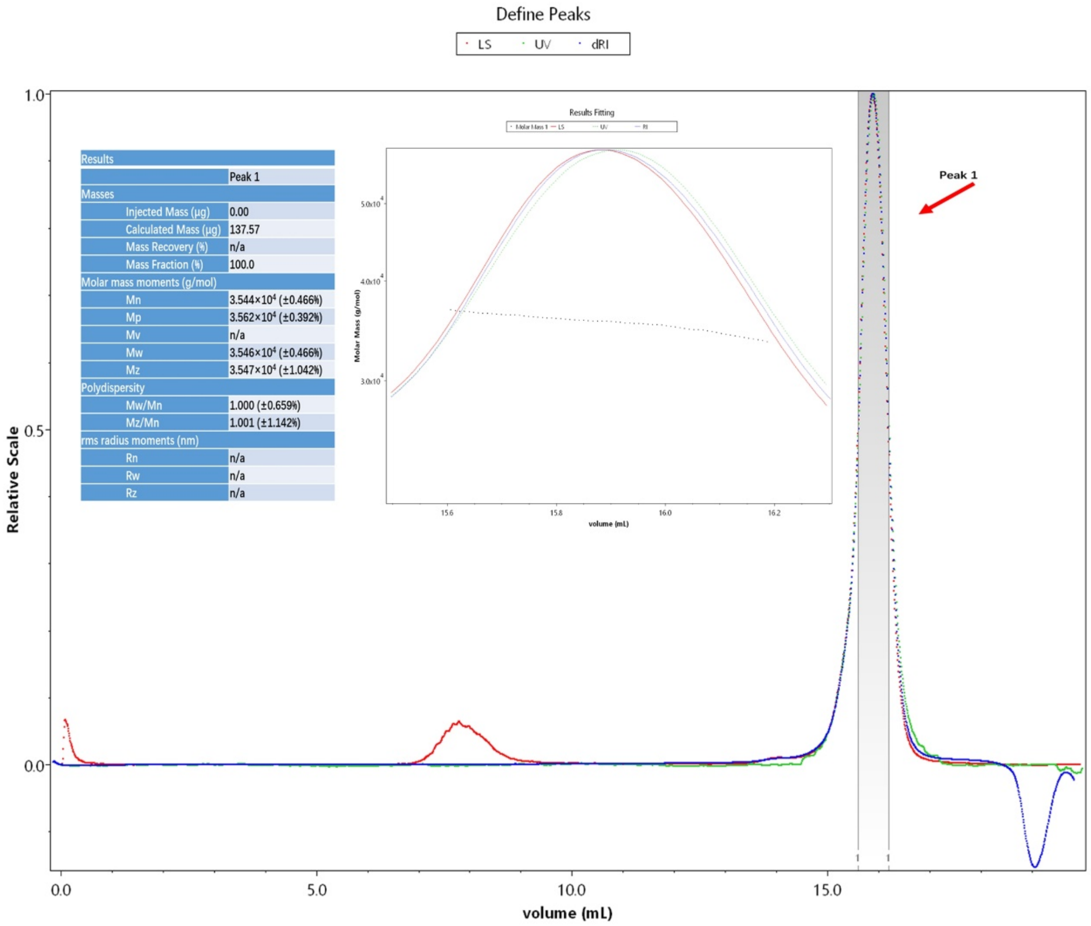

Supplementary Figure 1. Multi-angle light scattering of MCR-1C. Red arrow showed peak 1 of MCR-1C, and the insert table listed the parameters for peak 1. The average molecular weight is about 35.46kD, which suggests MCR-1C to be monomer.
